# Chronic Ethanol Causes Persistent Increases in Alzheimer’s Tau Pathology in Female 3xTg-AD Mice: A Potential Role for Lysosomal Impairment

**DOI:** 10.3389/fnbeh.2022.886634

**Published:** 2022-05-11

**Authors:** Autumn E. Tucker, Coral del Mar Alicea Pauneto, Alexandra M. Barnett, Leon G. Coleman

**Affiliations:** ^1^College of Arts and Sciences, University of North Carolina at Chapel Hill, Chapel Hill, NC, United States; ^2^Department of Pharmacology, University of North Carolina at Chapel Hill School of Medicine, Chapel Hill, NC, United States; ^3^Bowles Center for Alcohol Studies, University of North Carolina at Chapel Hill School of Medicine, Chapel Hill, NC, United States

**Keywords:** alcohol, Alzheimer’s disease, neuro-inflammation, abstinence, tau, addiction

## Abstract

Epidemiological studies have found that heavy alcohol use is associated with increased risk for Alzheimer’s disease (AD), with frequent drinking earlier in adulthood increasing risk. The increases in neuroinflammation featured in both heavy alcohol use and AD may be partially responsible for this link. However, it is unknown if abstinence mitigates this risk. We hypothesized that binge ethanol during mid adult life would persistently increase AD pathology even after prolonged abstinence. Male and female 3xTg-AD mice (APPSwe, tauP301, Psen1^tm1Mpm^) which feature progressive amyloid (Aβ) and tau pathology, received chronic binge ethanol (5g/kg/day, 5-days-on/2-days-off, i.g.) or water during adulthood (from 5.5 to 9 months of age), followed by abstinence and assessment at 14 months of age. The effects of ethanol on protective AD genes (e.g., APOE and TREM2) as well as proinflammatory genes were measured by PCR. Levels of pathologic tau and Aβ were measured by immunohistochemistry and western blot. Ethanol caused persistent reductions in protective AD genes: APOE (25% reduction, *p < 0.05), TREM2 (28%, *p < 0.05), LPL (40%, ^**^p < 0.01), and CTSD (24%, *p < 0.05) and promoted a proinflammatory gene signature in female, but not male cortex. Concurrently, ethanol increased total and hyperphosphorylated tau (AT8) in piriform cortex and hippocampus of females, but not males. Levels of AT8 were negatively correlated with APOE (*R* = –0.67, *p < 0.05) and TREM2 (*R* = –0.78, ^**^p < 0.005) suggesting protective roles in pathogenesis. No differences were found in levels of main regulators of tau phosphorylation state (GSK3β, PKA, PP2A), suggesting ethanol disrupted clearance of tau. Therefore, we measured the effect of ethanol on lysosomes, which degrade tau, and lysosomal localization of tau using co-immunofluorescence. In females, ethanol caused a persistent reduction in mature LAMP1 lysosomes in CA1 of hippocampus (35%, *p < 0.05), along with a 60% increase in total tau (*p < 0.05). Thus, chronic binge ethanol during mid adult life causes a persistent enhancement of tau pathology in cortical and hippocampal brain regions of females. Persistent AD pathology was associated with an increased proinflammatory signature and a reduction of mature lysosomes. This implicates binge ethanol exposure with increased risk of AD pathologic progression in females.

## Introduction

Heavy alcohol use has a strong historical and sociocultural presence globally but is also thought to be a causal factor in over 200 chronic diseases and injury-related conditions (Poznyak and Rekve, 2018). Nearly 1 in 3 American adults drinks alcohol excessively, with 25% reporting recent binge levels of alcohol use (Samhsa, 2020). Among the diverse effects of excessive drinking, heavy and binge alcohol use cause neurodegeneration (Crews and Vetreno, 2014; Coleman et al., 2017) and are increasingly being recognized as risk factors for chronic neurodegenerative disorders such as Alzheimer’s disease (AD) (Saunders et al., 1991; Dufouil et al., 2000; Mukamal et al., 2003; Jarvenpaa et al., 2005; Harwood et al., 2010; Langballe et al., 2015; Schwarzinger et al., 2018; Koch et al., 2019; Rehm et al., 2019). In fact, a large retrospective study with over a million subjects recently identified heavy alcohol use as the number one modifiable risk factor for dementia (Schwarzinger et al., 2018). Furthermore, a recent study has found that heavy drinking during early adulthood to midlife years was associated with increased risk for AD (Langballe et al., 2015). The majority of individuals that binge-drink are in late adolescent to mid-life years (18–44 yo) and “age-out” of heavy drinking, with low rates in aged populations (Bohm et al., 2021). This suggests that these epidemiological findings in humans could be due to persistent effects of binge ethanol during young adulthood to midlife years that increase risk for AD. Therefore, studies are needed to investigate the persistent impact of binge ethanol exposure during early to mid-adulthood on AD pathology later in life. Previous studies in rodents support this idea. Rodent studies often find that binge ethanol exposure earlier in life has long-term effects on neuronal populations that are lost early in AD (Coleman et al., 2011; Crews et al., 2021a). In humans, challenges exist in obtaining accurate self-reporting of lifetime alcohol use in patients with AD. However, a recent long-term prospective study (the HUNT trial) found that frequent alcohol use during midlife increased risk for AD dementia (Langballe et al., 2015). This suggests a long-term association between heavy alcohol use early in life with AD later in life.

AD is a progressive neurodegenerative disease characterized by memory loss, personality changes, and cognitive impairment. The classic hallmarks of Alzheimer’s disease are the accumulation of amyloid beta (Aβ) plaques and neurofibrillary tau tangles with progressive synaptic dysfunction and neuronal loss (Shi and Holtzman, 2018). Tau is a microtubule-associated protein that becomes phosphorylated at multiple residues during the progression of AD. However, recently neuroinflammation has been implicated as a likely contributor to AD pathogenesis (Zhang et al., 2013; Heneka et al., 2015; Dani et al., 2018; Jack et al., 2018; Shi and Holtzman, 2018; Long and Holtzman, 2019). Dysregulation of neuroimmune signaling may precede the development of AD pathology (Jack et al., 2018; Shi and Holtzman, 2018; Long and Holtzman, 2019). Many of the key proinflammatory molecules and cell types activated in AD are also induced by chronic alcohol use (He and Crews, 2008; Combs, 2009; Crews et al., 2013; Vetreno et al., 2013; Wu et al., 2015; Fujita et al., 2016). In AD proinflammatory activation can promote pathology (Sheng et al., 2003; Lee et al., 2008; Lee et al., 2010). However, microglia in AD mouse models also adopt protective disease-associated microglial (DAM) phenotypes as pathology progresses with phagocytic gene expression (Keren-Shaul et al., 2017), Neuroinflammation can be persistently induced by binge alcohol consumption (Vetreno and Crews, 2012; Coleman et al., 2021), which may suggest that binge ethanol during early life could promote future AD pathology (Venkataraman et al., 2017). Binge ethanol can also disrupt machinery involved in the degradation of pathologic amyloid and tau species. Pathologic tau species can be degraded by the ubiquitin-proteasome system (UPS), macroautophagy or chaperone-mediated autophagy (CMA) (Wang et al., 2010; Tang et al., 2019). Both macroautophagy and CMA culminate with degradation of tau in the lysosome. Alcohol can modulate autophagy in neurons (Luo, 2014) and binge ethanol early in life can persistently impair both the UPS and autophagy in adult brain (Pla et al., 2014). Thus, both neuroimmune and autophagic-lysosomal systems are disrupted by chronic binge ethanol and could converge to promote AD pathology later in life.

In this study we investigated the long-term, persistent effects of chronic binge ethanol on AD pathology later in adulthood. We used the triple-transgenic AD mouse model (3xTg-AD) that features three mutant human transgenes (APP^Swe^, tauP301, PSEN^tm1Mpm^) associated with human AD (Oddo et al., 2003). The 3xTg-AD mouse features progressive accumulation of Aβ and tau pathology during adulthood, beginning at 6 months (Belfiore et al., 2019). Aβ and tau pathology increase in cortical and hippocampal regions from 6 months (∼2.5% maximum) to 12 months (∼25% maximum), before reaching its maximum around 20 months (Belfiore et al., 2019). Mice were treated during midlife, during early development of AD pathology. Thus, mice received either binge ethanol or water from 5.5 to 9 months of age, followed by prolonged abstinence with assessment later in adulthood prior to maximal pathology (14 months). Given aforementioned findings in humans from the HUNT trial (Langballe et al., 2015), we hypothesized that chronic binge ethanol would promote AD pathology in 3xTg-AD mice even after prolonged abstinence. Further, we hypothesized ethanol would persistently increase pro-inflammatory signaling in brain and disrupt lysosomal function to promote AD pathology.

## Materials and Methods

### Animals

3xTg-AD breeders (APPSwe, tauP301, and Psen1^tm1Mpm^, Jackson Lab) were obtained through the Mutant Mouse Resource & Research Centers (MMRC). A homozygous breeder pair strategy was employed, with pups weaned by P30. All animal experiments were approved by the University of North Carolina at Chapel Hill Institutional Animal Care and Use Committee (IACUC) and were in accordance with NIH regulations (Protocols 20-232.0 and 21-052.0).

### Chronic Ethanol Treatment Paradigm

**FIGURE 1:**
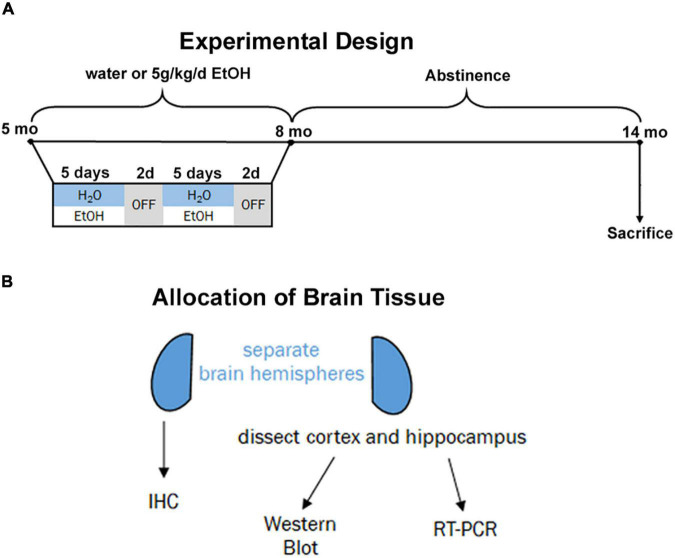
Experimental design and tissue allocation. **(A)** Adult 3xTg mice (5 mo, P168) received either water gavage (i.g.) or ethanol (5g/kg/d, i.g.) for 3 months (P168–P264), in a 5-days on 2-days off pattern. Mice were then left without intervention until sacrifice at 14 months (P432). **(B)** 1 hemisphere was drop-fixed in PFA for immunohistochemistry and 1 hemisphere dissected for cortex and hippocampus for both western blot and RT-PCR analyses.

Beginning at postnatal day 168 (P168), adult male and female 3xTg-AD mice received ethanol (5.0 g/kg, 20% ethanol, w/v, i.g., *N* = 3 males and 8 females) or an equal volume of water (*N* = 3 males, 4 females) based on weekly recorded weights. This dose produces blood alcohol concentrations of ∼250 mg/dL (Qin et al., 2007; Walter and Crews, 2017). Intragastric dosing was performed on a five-days-on/two-days-off schedule for three months (Figure 1A, P168–264). Both ethanol-treated mice and water-treated controls stopped receiving intragastric doses at P264 and remained under standard care after the final treatment without ethanol until being sacrificed at 14 months (P432). This experimental design was chosen to model heavy drinking in mid-life during a time when AD pathology is beginning to emerge the mouse model. All subjects were anesthetized with a euthanasia dose of sodium pentobarbital followed by transcardial perfusion with 0.1 M phosphate-buffered saline (PBS). Brains were excised and hemispheres separated.

### Tissue Storage, Preparation, and Usage

For each subject, one hemisphere was designated for immunohistochemistry (IHC) and the other to be used for RT-PCR and Western Blotting (Figure 1B). The hemisphere for IHC was drop-fixed in 4.0% paraformaldehyde at 4°C for at least 4 days followed by dehydration in 30% sucrose solution. Coronal sections (40 μm) were prepared using a sliding microtome (MICROM HM450) and stored at –20°C in cryoprotectant (30% glycol/30% ethylene glycol) until use. For the other hemisphere, the cortex and hippocampus were dissected and immediately frozen in liquid nitrogen.

#### Real-Time Polymerase Chain Reaction

Stored tissue samples were placed in RNase Free vials, lysed using Trizol (Invitrogen, Carlsbad, CA, United States), and homogenized by sonication. RNA was isolated by chloroform extraction as we have reported previously (Coleman et al., 2020; Qin et al., 2021; Crews et al., 2021b). After addition of chloroform, contents were mixed on a table rocker for 10 min at room temperature. Tubes were then spun at 13,000 rpm for 15 min at 4°C, and the aqueous (RNA-containing) phase was isolated. An equal volume of 100% isopropanol was added to each suspension, followed by mixing on a table rocker for 10 min and isolation of the RNA precipitate by centrifugation at 13,000 rpm for 15 min at 4°C. The RNA pellet was rinsed twice using cold 75% EtOH then spun at 9,000 rpm for 7 min at 4°C. The contents were dried for 10 min at room temperature before being re-suspended in DEPC-treated water. The RNA concentration was measured using a Nanodrop (Thermo Scientific, Wilmington, DE, United States) and was reverse transcribed to cDNA as we have done previously (Coleman et al., 2020; Qin et al., 2021; Crews et al., 2021b). The SYBR green PCR master mix (Applied Biosystems, Foster City, CA, United States) was used for real-time (RT) PCR analysis, which was performed by the CFX96 Real-Time System software. The relative differences in expression between groups were measured using cycle threshold (Ct) values normalized with β-actin, using the ddCt method to calculate the relative fold changes in gene expression. Primers used are listed in Table 1.

**TABLE 1:** Primers.

Gene	Forward (5’-3’)	Reverse (5’-3’)
IFNα	TACTCAGCAGACCTTGAACCT	CAGTCTTGGCAGCAAGTTGAC
IFNβ	TGGGTGGAATGAGACTATTGTTG	CTCCCACGTCAATCTTTCCTC
IFNγ	ATCTGGAGGAACTGGCAAAA	TTCAAGACTTCAAAGAGTCTGAGG
IL-6	GGCCTTCCCTACTTCACAAG	ATTTCCACGATTTCCCAGAG
TNF	GACCCTCACACTCAGATCATCTTCT	CCTCCACTTGGTGGTTTGCT
IL-1β	CTGGTGTGTGACGTTCCCATTA	CCGACAGCACGAGGCTTT
TLR4	GCCTTTCAGGGAATTAAGCTCC	AGATCAACCGATGGACGTGTAA
TLR7	ATGTGGACACGGAAGAGACAA	GGTAAGGGTAAGATTGGTGGTG
β2M	TTCTGGTGCTTGTCTCACTGA	CAGTATGTTCGGCTTCCCATTC
LPL	GGGAGTTTGGCTCCAGAGTTT	TGTGTCTTCAGGGGTCCTTAG
TREM2	CTCCAGGAATCAAGAGACCTCC	CCGGGTCCAGTGAGGATCT
APOE	CTCCCAAGTCACACAAGAACTG	CCAGCTCCTTTTTGTAAGCCTTT
Ctsd	GCTTCCGGTCTTTGACAACCT	CACCAAGCATTAGTTCTCCTCC
CD68	TGTCTGATCTTGCTAGGACCG	GAGAGTAACGGCCTTTTTGTGA
TGFβ	CTCCCGTGGCTTCTAGTGC	GCCTTAGTTTGGACAGGATCTG
IRF-3	AACCGGAAAGAAGTGTTGCG	CCCTGGAGTCACAAACTCATAC
TRAIL	GGGAGTCCTCTCGGAAAGG	CCGGATAGCTGGTGTACTTGTA
Caspase 3	ATGGAGAACAACAAAACCTCAGT	TTGCTCCCATGTATGGTCTTTAC
Caspase 8	TGCTTGGACTACATCCCACAC	TGCAGTCTAGGAAGTTGACCA
β-Actin (control)	CATTGCTGACAGGATGCAGAAGG	TGCTGGAAGGTGGACAGTGAGG

#### Western Blotting

Western blot was performed as we have reported previously (Coleman et al., 2017, 2018a). Briefly, frozen tissue samples were homogenized in lysis buffer (Tris–HCl, pH 7.5, Sucrose, EDTA, EGTA, 1% Triton X-100, protease, and phosphatase inhibitors). Protein concentration was measured by BCA assay. 40 μg of protein was run in each lane on SDS polyacrylamide gels followed by transfer to PVDF membranes. Membranes were washed in TBS and blocked for 1 h at room temperature in Li-Cor Blocking Solution (Li-Cor, Lincoln, NE, United States) then were incubated overnight at 4°C with primary antibody. The primary antibodies used for Western Blotting and their corresponding dilutions are listed in Table 2.

**TABLE 2:** Primary antibodies.

Primary antibody	Method	Dilution	Manufacturer (Catalog #)
Total Tau (Tau-5)	WB	1:1,000	Abcam (#ab80579)
Total Tau (Tau-5)	IF	1:250	Abcam (#ab80579)
Phosphorylated-tau214 (p-tau214)	WB	1:500	Invitrogen (#44-742G)
Beta-amyloid_1–42_ (Aβ_1–42_)	WB/IHC	1:1,000	Abcam (#ab201060)
Total GSK3β	WB	1:1,000	Cell Signaling (#12456)
Phosphorylated GSK3β (p-GSK3β)	WB	1:1,000	Invitrogen (#PA5-36646)
Protein Kinase A (PKA)	WB	1:1,000	Cell Signaling (#4781)
Protein Phosphatase 2A (PP2A)	WB	1:5,000	Abcam (#ab32104)
Phospho-Tau (Ser202, Thr205) (AT8)	IHC	1:100	Thermofisher (#MN1020)
Lamp1	IF	1:500	Abcam (#ab24170)
GAPDH (control)	WB	1:2,000	Cell Signaling (#97166)
			

Membranes were washed in TBS with 0.1% Tween-20 (Sigma-Aldrich, St. Louis, MO, United States) then were incubated in fluorescently conjugated secondary antibody (Rockland H&L, Limerick, PA, United States) at room temperature at the dilutions outlined in Table 3. Membranes were washed again in TBS then visualized using LiCor Image Studio Lite Ver 5.2 (either on the 700 or 800 channel). Western Blots were analyzed using Image Studio software. To account for any differences in protein loading in each well the level of the protein of interest was normalized to the level of the housekeeping protein GAPDH. Membranes were probed sequentially, for the protein of interest then for GAPDH the next day (or vice versa). All western blots for proteins that showed significant changes we replicated one to three times. Data are presented as the percent change relative to controls, averaged for each sample across all experimental replicates.

**TABLE 3:** Secondary antibodies.

Secondary antibody	Dilution	Manufacturer
Donkey anti-rabbit IgG	1:2,000	Rockland (#611-744-127)
Donkey anti-mouse IgG	1:2,000	Rockland (#610-745-124)
Goat anti-rabbit IgG	1:200	Vector Laboratories (#BA-1000)
Goat anti-rabbit IgG Alexa Fluor (594 nm)	1:1,000	Invitrogen (#A11012)
Goat anti-mouse IgG Alexa Fluor (488 nm)	1:1,000	Invitrogen (#A11001)

#### Immunohistochemistry and Immunofluorescence

IHC and IF were performed using techniques routinely used in our laboratory (Coleman et al., 2011, 2017, 2018b). Briefly, coronal sections were washed in PBS, then incubated for 1 hour at 70°C for antigen retrieval in Citra solution (BioGenex, Freemont, CA, United States). Endogenous peroxidase activity was removed by incubation in 0.6% H_2_O_2_ for 30 min. Sections were washed and nonspecific binding blocked by incubation in PBS with 4% serum (species of the secondary antibody) and 0.1% TX-100. Sections were incubated in primary antibody (in blocking buffer) overnight at 4°C, using the dilutions indicated in Table 2. The following day sections were washed, then incubated with the appropriate secondary antibody, listed in Table 3. The avidin-biotin (ABC) method (Vectastain Elite Kit, Vector Labs; Burlingame, CA, United States) was used to visualize immunolabeling, along with diaminobenzidine (DAB) and nickel enhancement which was applied to all sections simultaneously for the same period of time. For IF, sections were placed in blocking buffer after antigen retrieval, with visualization on day 2 with the appropriate fluorescent secondary antibody. Sections for IF were mounted and cover-slipped with anti-fade mounting medium (Thermofisher, Waltham, MA, United States). Images of slides and staining were gathered using the BZ-X180 fluorescent microscope and accompanying software (Keyence, Ithaca, NY, United States). Quantifications were measured on and collected from the ImageJ software (NIH, Bethesda, MA, United States).

### Statistical Analysis

For preplanned orthogonal comparisons between two groups *t*-tests were used. Data is reported as mean ± standard error of the mean (SEM) and a *p* value of less than 0.05 was considered significant. All data analysis was conducted using the GraphPad Prism 9 software. Significant outliers were detected using the Grubb’s test for outliers (GraphPad). For proinflammatory gene analysis Gene Set Enrichment Analysis (GSEA) was performed using the GSEA software (Mootha et al., 2003; Subramanian et al., 2005) and the Hallmark Inflammatory Response gene set from the Molecular signatures database (Liberzon et al., 2015). Significant enrichment was determined by a nominal *p* value < 0.01.

## Results

### Ethanol Persistently Blunted Expression of Protective Microglial Genes and Caused Proinflammatory Activation in Females 3xTg-AD Mice

**FIGURE 2:**
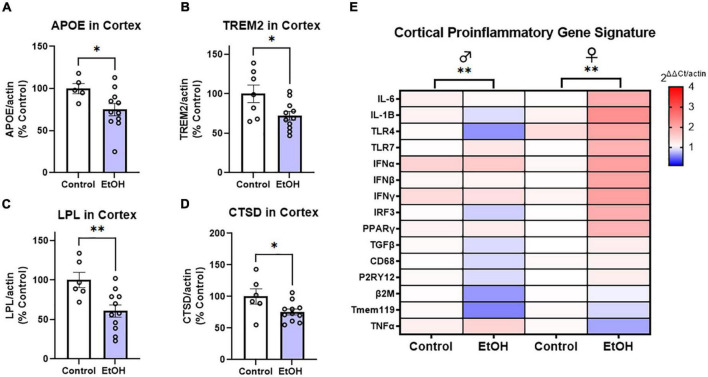
Chronic binge ethanol persistently reduces key disease-associated microglial (DAM) genes and promotes a proinflammatory gene profile in females. Adult male and female 3xTg-AD mice received EtOH (5g/kg/d, i.g., 5d on 2d off) for 3 months (P168–P264), followed by prolonged abstinence until 14 months of age. Gene expression was measured by RT-PCR. **(A–D)** Expression of key protective DAM genes in cortex of both males and females (combined). Ethanol caused a significant reduction of **(A)** APOE, 25%, **(B)** TREM2, 28%, **(C)** LPL, 40%, and **(D)** CTSD, 24%. *p < 0.05, ^**^p < 0.01, *t-*tests. **(E)** Heat map showing expression of pro-inflammatory genes in cortex. Ethanol persistently increased expression of several proinflammatory genes in females (11/13), with reductions in several genes found in males (8/13 genes). Gene Set Enrichment Analysis (GSEA) found significant enrichment of genes in both sexes (ethanol vs control), *N* = 3 males/group; 3 female control and 8 female ethanol. ^**^p < 0.01.

To determine if ethanol exposure during early adulthood promotes pathology later in life, mice received binge ethanol treatment beginning at 5.5 months, prior to the emergence of notable pathology. Mice received ethanol for 3 months as Aβ and tau increased followed by prolonged abstinence and assessment at 14 months of age (Figure 1A). Neuroimmune dysregulation occurs in both AD and binge ethanol. Therefore, we first examined the persistent effect of ethanol on key DAM genes and proinflammatory genes in the cortex. APOE and TREM2 are critical genes associated with risk for AD. In humans dysfunctional APOE4ε isoforms confer increased risk for AD while upregulation of TREM2 reduces pathology in AD mouse models (Strittmatter et al., 1993; Corder et al., 1994; Jiang et al., 2014). Ethanol persistently reduced expression of both APOE (Figure 2A, 25%, *p < 0.05) and TREM2 (Figure 2B, 28%, *p < 0.05). Lipoprotein lipase (LPL) is a critical regulator of lipid uptake and metabolism that is increased in phagocytic microglia in AD models and humans with AD (Keren-Shaul et al., 2017). Ethanol caused a persistent reduction in expression of LPL (Figure 2C, 40%, ^**^p < 0.01). Cathepsin D (CTSD), a key lysosomal protease was also reduced by ethanol (Figure 2D, 24%, *p < 0.05). Since no differences were found in DAM gene expression between males and females, sexes were combined. However, a significant sexual dimorphism was seen in pro-inflammatory gene expression in the cortex. In general, ethanol increased proinflammatory gene expression profile in 3xTg female, but not male mice (Figure 2E). GSEA using the Hallmark Inflammatory Response gene set found significant core enrichment (*p <* 0.01) in response to ethanol treatment in both males and females, with the majority of genes in males (8/13, Supplementary Table 1) having negative rank metric scores and 11/13 genes in the females with positive rank metric scores (Supplementary Table 2). Thus, ethanol persistently reduces expression of key DAM genes in both sexes, while inducing expression of a proinflammatory gene profile in female, but not male mice.

### Chronic Ethanol Causes Persistent Increases in Cortical and Hippocampal Tau Pathology in Female 3xTg-AD Mice

**FIGURE 3:**
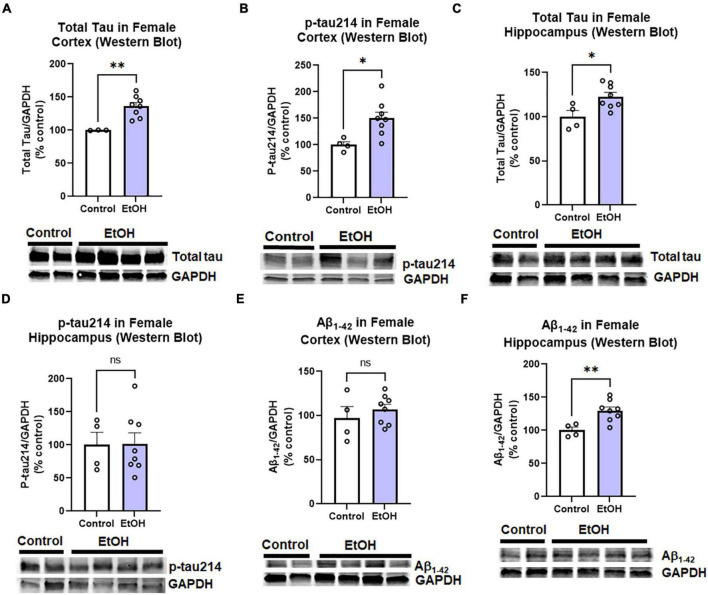
Persistent effects of ethanol on tau and Aβ proteins in female 3xTg-AD mice. Adult female 3xTg-AD mice received either EtOH (5g/kg/d, i.g., 5d on 2d off, *N* = 8) or the same schedule of water (i.g., *N* = 4) for 3 months (P168–P264), followed by prolonged abstinence until 14 months. Pathologic tau and Aβ protein levels were measured in cortex and hippocampus by western blot. **(A,B)** Tau species in cortex. In the cortex EtOH caused a persistent increase in **(A)** total tau, 36% (*N* = 3 control, 8 ethanol, 1 outlier) and **(B)** phosphorylated tau214, p-tau214, 50%, *p < 0.05. **(C,D)** Tau in hippocampus. In the hippocampus, EtOH persistently increased levels of **(C)** total Tau, 23% increase, *p < 0.05 without altering **(D)** p-tau214, *p* = 0.9 levels. **(E,F)** Aβ_1–42_ levels. EtOH had no persistent impact on levels of Aβ_1–42_ protein in female cortex, *p* = 0.4 **(E)**, though a **(F)** 30% increase was found in the hippocampus. *p < 0.05, **p < 0.01, *t-*test.

The observed effects of ethanol on protective and proinflammatory genes above suggested ethanol might also increase AD protein pathology. This includes both pathologic amyloid Aβ_1–42_ and hyperphosphorylated tau species. Tau phosphorylated at serine residue 214 is an AD-specific phosphorylation site (Li and Gotz, 2017). Therefore, we measured levels of total tau, phosphorylated tau-214 (p-tau214), and Aβ_1–42_ in cortex and hippocampus by Western blot. Similar to findings in humans, where females have a 2-fold greater risk for AD than males (Seshadri et al., 1997), we observed a sexual dimorphism. In female cortex, there were significant increases in levels of total tau (Figure 3A, 36%, ^**^p < 0.01) and p-tau214 (Figure 3B, 50%, *p < 0.05) as a result of prior chronic ethanol consumption. In female hippocampus total tau was also increased (Figure 3C, 23%, ^**^p < 0.01) though no difference in p-tau214 was observed (Figure 3D). Regarding Aβ_1–42_ levels, no change was found in the cortex (Figure 3E), however a 30% increase was found in the hippocampus (Figure 3F, *p < 0.05). To ensure measured changes in protein expression were not due to an effect of ethanol on levels of the housekeeping protein, we assessed the effect of ethanol on GAPDH. We found that ethanol had no significant effect on GAPDH in either males or females. Ethanol did not increase expression of the tau transgenes microtubule-associated protein tau (MAPT) in the cortex (data not shown, *p* = 0.33) or hippocampus (data not shown, *p* = 0.45). Expression of amyloid precursor protein (APP) was also unchanged by ethanol in the cortex (data not shown, *p* = 0.34) and hippocampus (data not shown, *p* = 0.78). We also assessed the persistent effect of ethanol on neurotoxicity as this could contribute to increases in AD pathology. Intrinsic cell death has been implicated in both AD and alcohol with mediators such as TRAIL and caspase-3 being upregulated (Burgaletto et al., 2020; Qin et al., 2021). Ethanol did not persistently induce expression of caspase-3, caspase-8 or TRAIL in cortex (Supplementary Figure 1). Thus, binge ethanol persistently increases protein levels tau and Aβ_1–42_ in the aged 3xTg-AD female cortex and hippocampus, without changing their gene expression.

**FIGURE 4:**
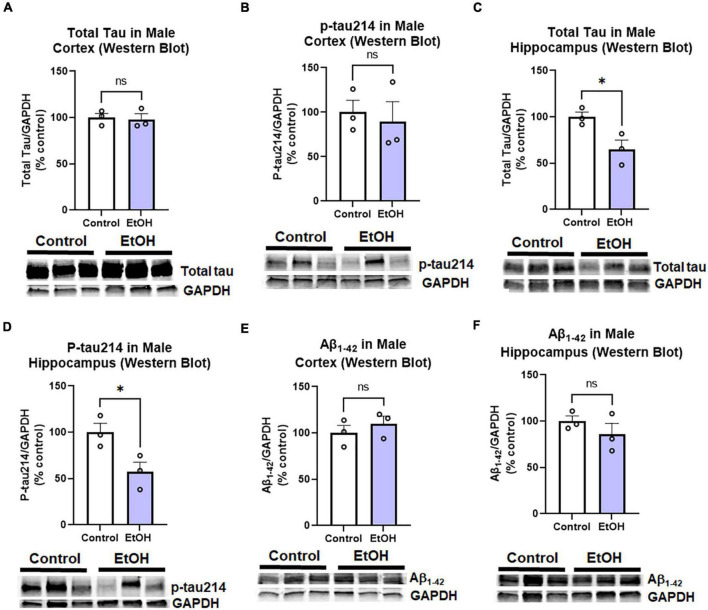
Persistent effects of ethanol on tau and Aβ proteins in male 3xTg-AD mice. Adult male 3xTg-AD mice received EtOH (5g/kg/d, i.g., 5d on 2d off, *N* = 3) or the same schedule of water (i.g., *N* = 3) for 3 months (P168–P264), followed by prolonged abstinence with assessment at 14 months. Pathologic tau and Aβ protein levels were measured in cortex and hippocampus by western blot. **(A,B)** Tau species in cortex. EtOH had no persistent effects on neither **(A)** total tau, *p* = 0.8 nor **(B)** phosphorylated tau214 (p-tau214) *p* = 0.7. **(C,D)** Tau in hippocampus. In the hippocampus, EtOH caused a persistent reduction in **(C)** total Tau, 35% reduction, *p < 0.05, and **(D)** p-tau214, 43% reduction, *p < 0.05. **(E,F)** Aβ_1–42_ levels. EtOH had no persistent impact on levels of Aβ_1–42_ in either male **(E)** cortex, *p* = 0.4 or **(F)** hippocampus, *p* = 0.3. *p < 0.05, *t-*test.

**FIGURE 5:**
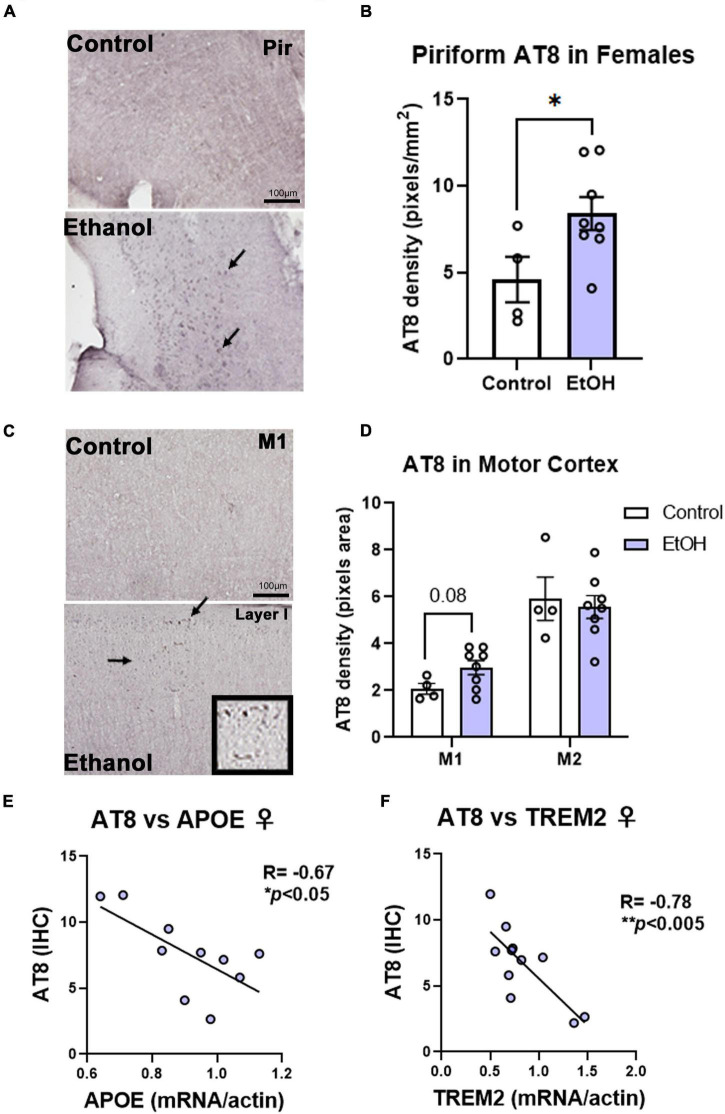
Chronic binge ethanol persistently increases phosphorylated tau AT8 staining in female cortex. Adult female 3xTg-AD mice received EtOH (5g/kg/d, i.g., 5d on 2d off) for 3 months (P168–P264), followed by prolonged abstinence at assessment at 14 months. Pathological phosphorylated tau (AT8) was assessed by IHC in piriform and motor cortex. **(A)** Representative images showing increased AT8 immunoreactivity (+IR) in piriform cortex caused by ethanol. Arrows denote AT8+IR cells. **(B)** Quantification of AT8 pixel density showed a ∼2-fold increase in AT8 in the piriform cortex after ethanol. *p < 0.05, *t-*test. **(C)** Images of M1 motor cortex showing low levels of AT8 in control mice, but slightly increased +IR was seen in several ethanol-treated mice. High magnification insert shows cells positive for AT8. **(D)** Quantification showed a trend toward a 33% increase in AT8 in M1 of ethanol-treated mice (*p* = 0.08, *t*-test). No differences were seen in AT8 +IR in M2 of the motor cortex, though higher baseline levels were seen than in M1. *p < 0.05, *t-*test. **(E)** AT8+IR was negatively correlated with cortical APOE mRNA levels. *R* = –0.67, *p < 0.05, Pearson’s correlation coefficient. **(F)** AT8 +IR was negatively correlated with gene expression of TREM2 in cortex. *R* = –0.78, *N* = 4 control, 8 ethanol. ^**^p < 0.005.

Contrary to females, chronic ethanol did not have significant detrimental effects on AD protein pathology in male mice. There were no persistent increases in total tau (Figure 4A) or p-tau214 (Figure 4B) in the cortex. Surprisingly, in the hippocampus there were reductions in both total tau (Figure 4C, 35%, *p < 0.05) and p-tau214 (Figure 4D, 43%, *p < 0.05). Further, there were no persistent alcohol effects on Aβ_1–42_ levels in either cortex (Figure 4E) or hippocampus (Figure 4F). Given the persistent increases in tau protein levels in the cortex of female subjects by western blot, we performed IHC to visualize pathologic tau across brain regions. AT8, a marker of tau hyper-phosphorylation (Ser202/Thr205), was assessed by IHC. Increased staining for AT8 was noticed in the piriform (Figure 5A) and motor cortex (Figure 5C). Quantification revealed a 2-fold increase in AT8 immunoreactivity (+IR) in the piriform cortex following alcohol treatment (Figures 5A,B, *p < 0.05). Investigation of the motor cortex found a trend toward a 33% increase in AT8 +IR in M1 (Figure 5C, *p* = 0.08), with no persistent differences in M2 (Figure 5D). Given that we found a reduction in protective genes APOE and TREM2 above, we assessed if AT8 in the piriform cortex was associated with their gene expression levels. Consistent with a protective role of APOE and TREM2, we found strong negative correlations of AT8 with APOE (Figure 5E, *R* = –0.67, *p < 0.05) and TREM2 (Figure 5F, *R* = –0.78, **p < 0.005). Thus, ethanol persistently increases levels of hyperphosphorylated tau in cortical brain regions of female 3xTg-AD mice.

### Effects of Chronic Ethanol on Autophagy and Lysosomal Tau Clearance in Female Subjects

**FIGURE 6:**
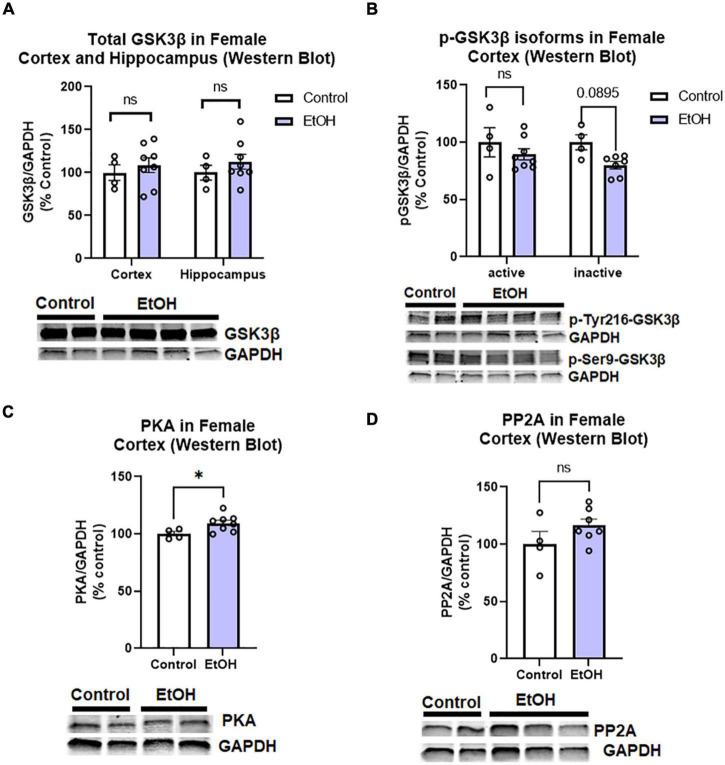
Chronic binge ethanol has no persistent effect on major regulators of tau phosphorylation in female 3x-TgAD mice. Adult female 3xTg-AD mice received EtOH (5g/kg/d, i.g., 5d on 2d off) for 3 months (P168–P264), followed by prolonged abstinence until 14 months. Common tau-phosphorylating kinases (GSK3β and PKA) and the tau de-phosphorylating enzyme PP2A were measured by Western Blot. **(A)** Total GSK3β protein level in cortex was unchanged by ethanol. **(B)** Levels of active p-Tyr216 GSK3β were unchanged by ethanol and levels of the inactive p-Ser9-GSK3β showed a trend toward a 20% reduction, *p* = 0.09. **(C)** Ethanol caused a slight 5% increase in protein kinase A (PKA), *p < 0.05. **(D)** Ethanol had no effect on expression levels of PP2A. *N* = 4 control, 8 ethanol. *p < 0.05, *t-*test.

Since we found increased levels of phosphorylated tau in females, we assessed if this could be attributed to main tau phosphorylating enzymes. Tau can be phosphorylated by many kinases and dephosphorylated by phosphatases (Li and Gotz, 2017). We assessed to two major tau phosphorylating kinases that are important in AD pathologic tau progression and are known to be induced by ethanol; glycogen synthase kinase 3 beta (GSK3β) and protein kinase A (PKA; Luo, 2009; Behl et al., 2021). We also measured protein phosphatase 2A (PP2A), which is responsible for ∼70% of tau dephosphorylation (Liu et al., 2005). We found that there were no persistent changes in total GSK3β in the hippocampus or cortex of female subjects, (Figure 6A) nor active (p-Tyr216) or inactive (pSer9) phosphorylated isoforms of GSK3β in the female cortex (Figure 6B). PKA levels were minimally increased in the cortex (Figure 6C, 5%, *p < 0.05), and there were no significant differences in PP2A levels following ethanol treatment in female subjects (Figure 6D). Despite increases to tau and p-tau levels in female subjects, there is an apparent lack of a persistent effects on the major tau phosphorylating enzymes and transgene expression to describe this difference. Therefore, we hypothesized that ethanol may be involved in a reduction or disruption of tau clearance.

**FIGURE 7:**
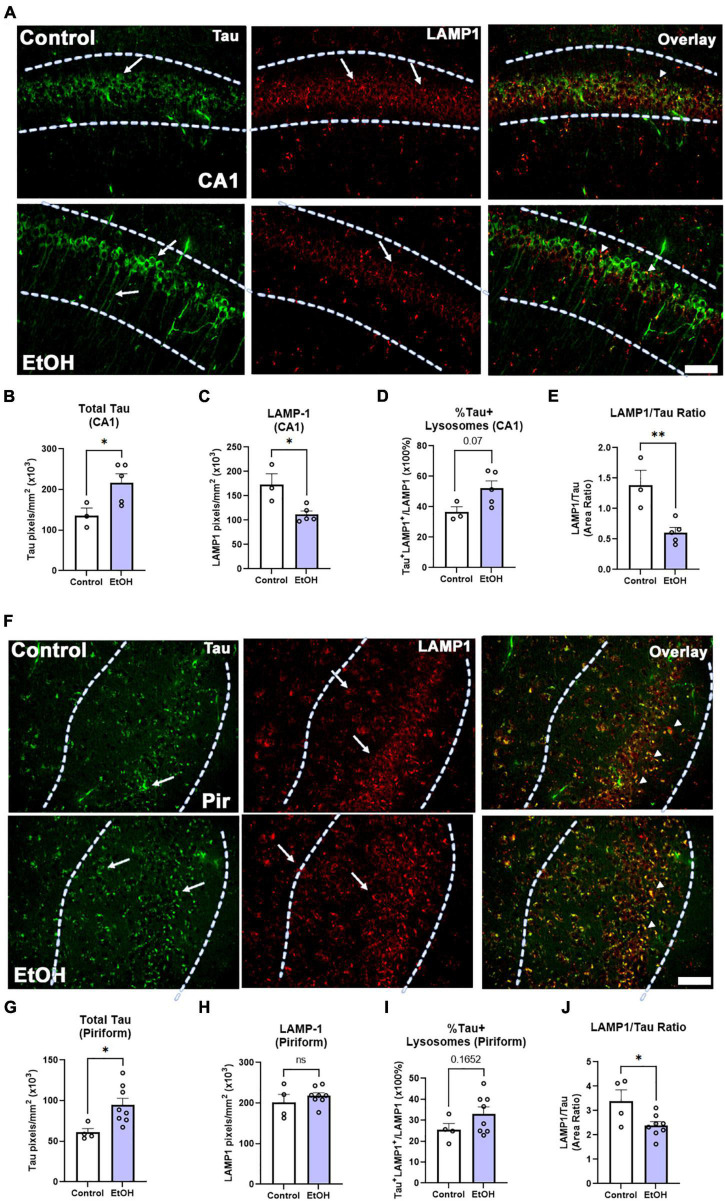
Chronic binge ethanol causes persistent increase in tau and loss of LAMP1 positive lysosomes in hippocampus of female 3xTg-AD mice. Adult female 3xTg-AD mice received EtOH (5g/kg/d, i.g., 5d on 2d off) for 3 months (P168–P264), followed by prolonged abstinence until 14 months. **(A)** Total tau and lysosomal LAMP1 involved in autophagy and tau clearance were measured by co-immunofluorescence (co-IF). Left-pointing arrows denote tau stain in cell body and processes. Right pointing arrows denote punctate LAMP-1 staining. Arrowheads denote co-localization. **(B)** Quantification of total tau in the CA1 region revealed a persistent increase by 60%, *p < 0.05, *t-*test and **(C)** quantification of lysosomal LAMP1 in the CA1 region indicated a persistent reduction by 35%, *p < 0.05. **(D)** Overlaying total tau and LAMP1 revealed a trend toward a 43% increase in the percent of tau-positive lysosomes in the CA1, *p* = 0.07, however, **(E)** there was also a sharp 59% decline in the amount of LAMP1 overlapping with total tau in the CA1, ^**^p < 0.01. **(F)** Total tau and lysosomal LAMP1 involved in autophagy and tau clearance were measured by co-IF in piriform cortex (Pir). Left-pointing arrows denote tau stain in cell body and processes. Right pointing arrows denote punctate LAMP-1 staining. Arrowheads denote co-localization. **(G)** Ethanol caused a persistent increase in tau in the piriform cortex (Pir), 56%m*p < 0.05, *t-*test. **(H)** Ethanol had no effect on LAMP1 level in Pir. **(I)** Ethanol caused a trend toward an increase in the percent of Tau+ lysosomes *p* = 0.17. **(J)** Ethanol caused a persistent reduction in the ratio of LAMP1 to Tau. *p < 0.05, *t-*test. Scale bar = 50μm.

Both macroautophagy and chaperone-mediated autophagy are key methods of tau clearance that both result in lysosomal degradation of tau. Binge ethanol early in life can persistently disrupt macroautophagy (Pla et al., 2014), therefore we hypothesized that chronic binge ethanol might persistently impair lysosomal clearance of tau. Thus, given the increased tau pathology in female subjects, we measured the ethanol effect on lysosomal proteins involved in tau clearance by autophagy. Lysosome-associated member protein 1 (LAMP1), a mature lysosomal marker, was fluorescently visualized in female subjects, along with tau-5, a marker for total tau (Figure 7A). Consistent with results reported in Figure 3C, there was a significant increase in total tau accumulation in the CA1 region of the hippocampus (Figure 7B, 60%. *p < 0.05). Additionally, there was a notable decrease in hippocampal LAMP1 levels following prior binge ethanol consumption (Figure 7C, 35%, *p = 0.05). Joint analysis of tau and LAMP1 levels in the CA1 further revealed a trend toward an increase in the percent of lysosomes containing tau (Figure 7D, 43%. *p* = 0.07) along with a sharp reduction in the ratio of LAMP1 overlapping with tau across the CA1 following ethanol treatment (Figure 7E, 56%, ^**^p < 0.01). This suggests that there is an insufficient number of lysosomes to account for the increased levels of tau. In the piriform cortex, tau was increased by ethanol to a similar extent as in the hippocampus (Figures 7F,G, 56%, *p < 0.05) though the baseline level of tau was 55% lower in the piriform than in the hippocampus, consistent with tau pathology arising earlier in the hippocampus than cortical brain regions(Belfiore et al., 2019). LAMP1 levels were unchanged in the piriform (Figure 7H), though a trend toward an increase in the percentage of tau-occupied lysosomes was found (Figure 7I, *p* = 0.17) and the LAMP1/tau ratio was reduced by 30% (Figure 7J, *p < 0.05). Thus, in the hippocampus, a region that develops tau pathology early, a reduction of LAMP1 was found along with increased tau, suggesting reduced lysosomal clearance of tau later in disease progression caused by ethanol use earlier in life.

## Discussion

Chronic heavy alcohol use is increasingly being recognized as a risk factor for Alzheimer’s disease and all cause dementia (Saunders et al., 1991; Mukamal et al., 2003; Harwood et al., 2010; Langballe et al., 2015; Schwarzinger et al., 2018; Koch et al., 2019; Rehm et al., 2019). In fact, binge alcohol and alcohol-related brain damage were recently found to be the number one modifiable risk factor for dementia (Schwarzinger et al., 2018), with frequent use during midlife increasing risk for AD (Langballe et al., 2015). Heavy alcohol and AD share several commonalities, such as proinflammatory microglial activation and neurodegeneration (Wang et al., 2015; Crews et al., 2017; Long and Holtzman, 2019). Findings here using a chronic binge ethanol exposure during midlife in 3xTg mice persistently increases tau pathology with aging, reducing levels of protective microglial DAM genes and causing proinflammatory activation in females. We found that total tau was increased in females as measured by both western blot and immunofluorescence. Further, phosphorylated tau species were found to be increased in female cortex by both western blot and IHC. Together, findings across these complementary approaches support true increases in tau species in females after ethanol. Ethanol also increased Aβ_1–42_ in female hippocampus. The cleaved Aβ_1–42_ peptide is neurotoxic and its accumulation is considered a precursor to plaque formation (Billings et al., 2005). Further, the data indicates that chronic ethanol persistently reduces lysosomal clearance of tau. Thus, consistent with epidemiological findings, this data indicates that excessive drinking in earlier adulthood could also have lingering effects that persist during abstinence into later adulthood.

It is important to note that the time frame of treatment models heavy drinking in young adulthood/mid-life, during a time in which AD pathology is beginning to emerge in this mouse model. However, it is difficult to exactly translate the ages in the 3xTg-AD for treatment with the natural history in humans. In the 3xTg-AD mouse, AD pathology begins to emerge between 6 and 12 months of age (Belfiore et al., 2019) which is considered young adulthood to early midlife in humans. Thus, we balance treating mice during a time associated with the early emergence of pathology with an age range near midlife in humans. Further, the time of assessment is when levels of AD pathology would be significant (∼50% maximal; Belfiore et al., 2019), but not at their ceiling when ethanol-related differences would not be able to be detected. In wild types this age corresponds to the ∼1950s to 1960s though the differential expression of the transgene makes this conversion difficult in the 3xTg-AD. Nonetheless, our findings suggest that binge ethanol exposure earlier in adulthood persistently increases AD pathology as the disease progresses. These findings, coupled with epidemiological data that finds increased risk with heavy drinking up to 27 years before AD diagnosis (Langballe et al., 2015) and the fact that most binge-drinkers “age out,” this suggests even 15–20 years of abstinence could still be detrimental to AD pathology.

We found that ethanol persistently reduced the expression of protective microglial DAM genes, such as APOE, TREM2, LPL, and CTSD. These molecules promote phagocytic capacity and are thought to be protective against the progression of AD pathology (Keren-Shaul et al., 2017). Though originally identified in microglia in a model of cerebral amyloid angiopathy (5XFAD), dysfunction of key genes we found reduced by ethanol (e.g. APOE and TREM2) are well known risk factors for AD in general, and are involved in lipid metabolism, phagocytosis, and are known to influence tau pathology (Guerreiro et al., 2013; Jiang et al., 2014; Carmona et al., 2018; Yamazaki et al., 2019; Lee et al., 2021). Further, levels of hyperphosphorylated tau were negatively correlated with expression of APOE and TREM2. At the same time, ethanol increased expression of proinflammatory cytokines in female cortex. Proinflammatory signaling in brain can accelerate AD amyloid and tau pathology (Sheng et al., 2003; Lee et al., 2008; Liu et al., 2016). However, gene expression levels often do not correlate with protein levels, which is a limitation of this study. Nevertheless, these findings suggest that chronic binge ethanol can persistently alter the proinflammatory/phagocytic milieu to promote progression of AD pathology. Future studies will investigate a causal link between these immune effects of ethanol and AD pathology. An additional future direction of this work is to assess the persistent impact of ethanol on behavioral functions, as this was not assessed. Other studies, however, report that ethanol-enhancement of AD pathology in AD mouse models can negatively impact behavior. A recent study in APP/PSE mice using a daily binge model ethanol (2.5g/kg, i.p.) during adolescence (P20–P60) found persistent increases in protein levels of Aβ42 in the hippocampus at 6 and 12 months of age and causes working memory deficits (Ledesma et al., 2021). Another study by Hoffman et al. (2019) using voluntary drinking (moderate exposure) in 10-week-old adult 3xTg-AD mice for 4 months showed increased levels of p-tau-199/202 in hippocampus and increased Aβ42/40 ratios in cortex that persisted one month into ethanol abstinence (7 months of age at assessment) with reduced memory retention in the probe trial of the Morris water maze.

A limitation of this work is that wild type mice were not assessed. Therefore, we cannot detect the ethanol × genotype interactions. The majority of wild-type mouse strains do not develop AD-like pathology. Accordingly, the aforementioned studies assessing the effect of ethanol on AD pathology did study wild type mice, finding no effects of ethanol on amyloid or tau in wild type mice (Hoffman et al., 2019; Ledesma et al., 2021). However, studies in adolescent wild type mice find ethanol does persistently promote proinflammatory signaling (Vetreno and Crews, 2012; Montesinos et al., 2015) though it is unclear if this occurs in adults. Further, we employed a non-contingent ethanol paradigm in which mice received either ethanol or water gavage. This was chosen to produce high BACs and reduce variability in blood alcohol levels between subjects. Hoffman et al. indicate that self-administration paradigms can also result in enhanced AD pathology in mouse models. However, future work should employ voluntary paradigms such as drinking in the dark, intermittent access or two-bottle choice.

In these studies, a clear sexual dimorphism was observed, with female subjects showing persistent reductions in protective microglial genes, increased proinflammatory activation, and increased AD protein pathology. For the most part, males showed no differences after ethanol treatment and abstinence. However, they did show reduced total and phosphorylated tau in the hippocampus. The underlying mechanism for this is unclear, but this will be examined further in future studies. In humans, females have a 2-fold increased risk for AD (Seshadri et al., 1997) and have more accelerated cognitive decline than males (Ferretti et al., 2018). The 3xTg-AD mouse shows a similar worsening of pathology and acceleration of cognitive decline in females (Billings et al., 2005; Carroll et al., 2010). Despite the clear sexual dimorphism in this disorder, little is known about the root of this sex-based difference, though sex hormones are thought to contribute (Carroll et al., 2010). Sexually dimorphic effects of heavy alcohol use on tissue injury are also known. Women who drink excessively have a higher risk for developing medical problems compared to their male counterparts (Erol and Karpyak, 2015). Women are more susceptible to alcohol-related liver inflammation, heart disease, and neurological problems such as blackouts and hangovers than men (White, 2020). Given that rates of binge drinking in women are increasing (Samhsa, 2020; Slade et al., 2016; White, 2020) and the increased risk of developing AD for women, these findings raise cause for concern and a need to better understand the underlying causes of the increased risk for AD pathology in women. Studies in periphery find that female mice have increased liver inflammation and damage with chronic binge consumption than males (Fulham and Mandrekar, 2016), and women have higher circulating levels of bacterial endotoxin than males after acute alcohol exposure (Bala et al., 2014). Regarding the persistent effects in brain; however, few studies have investigated sex differences in the persistent induction of neuroimmune genes in adults (Mineur et al., 2022). The majority of studies have been done in males, finding acute morphological and molecular activation of microglia and proinflammatory gene induction (for Review see (Mineur et al., 2022) and (Crews et al., 2017)). Some evidence does suggest that females might have greater microglial functional responses to binge ethanol than males. Barton et al. reported that four days of heavy binge ethanol increased the number of MHC II+ activated/hyper-ramified microglia in the mPFC and hippocampus of female but not male rats (Barton et al., 2017). Further, female human adolescents and young adults have increased levels of serum proinflammatory cytokines than males, and female mice have increased proinflammatory cytokines, TLR4 and proinflammatory NFκB 24 h after 8-doses of binge ethanol (3g/kg, i.p.) than males (Pascual et al., 2017). We recently reported using a similar adolescent treatment in 3xTg-AD mice (5g/kg/d 2-days on 2-days off, P25–P55) that females, but not males, showed persistent proinflammatory gene induction along with increased AD pathology in the hippocampus in adulthood (P200), that was prevented by the anti-inflammatory compound minocycline (Barnett et al., 2022). However, the question of the sex-differences in the persistence of proinflammatory signaling after ethanol during adulthood has not been rigorously assessed previously in wild-type adult rodents. This and other persistent sex-specific alterations are a critical investigations that should be pursued further in future studies.

Recently, lysosomal dysfunction has gained increased attention in the pathology of AD. Proper lysosomal function is necessary for clearance of both intracellular and extracellular tau and amyloid (Lai et al., 2021). Lysosomal dysfunction has also been implicated in the APOEε4 risk-genotype (Schmukler et al., 2018). In the hippocampus, we found a reduction of mature lysosomes along with an increase in tau accumulation. We did not find a change in lysosomes in the piriform, though increased tau was also seen. The hippocampus shows tau pathology earlier than other regions in the 3xTg-AD model (Belfiore et al., 2019). Thus, it is possible that the persistent increase of tau caused by ethanol results in a loss of lysosomes as the disease progresses, perhaps due to a persistent increase in lysosomal burden. This could, in turn, further promote pathology. The hippocampus is critical for learning and memory function and shows tau pathology early in human disease (Braak and Braak, 1991; Long and Holtzman, 2019). In cortex, we observed an increase in tau particularly in the piriform cortex. The piriform is particularly vulnerable to alcohol neurotoxicity, as is the hippocampus (Crews et al., 2000; Obernier et al., 2002a,b). The piriform plays a crucial role in olfactory processing and memory and shows dysfunction in AD (Ebadi et al., 2017; Yan et al., 2022). Deficits in olfaction occur early in AD, mirror tau accumulation, and precede memory deficits, and are considered to be a potential early diagnostic symptom (Devanand et al., 2015; Murphy, 2019). Thus, increased tau in the piriform cortex and hippocampus by ethanol could accelerate the progression of disease.

This work indicates that chronic binge ethanol during mid-adult life has long-term effects on AD pathology, particularly in females. Ethanol persistently increases tau and immune dysfunction in key regions involved in AD pathology. Future work will determine if reversing the proinflammatory activation caused by ethanol can prevent this persistent enhancement.

## Data Availability Statement

The original contributions presented in the study are included in the article/Supplementary Material, further inquiries can be directed to the corresponding author.

## Ethics Statement

The animal study was reviewed and approved by University of North Carolina at Chapel Hill Institutional Animal Care and Use Committee.

## Author Contributions

AT and LGC drafted the initial version of the manuscript. All authors approved the final version.

## Conflict of Interest

The authors declare that the research was conducted in the absence of any commercial or financial relationships that could be construed as a potential conflict of interest.

## Publisher’s Note

All claims expressed in this article are solely those of the authors and do not necessarily represent those of their affiliated organizations, or those of the publisher, the editors and the reviewers. Any product that may be evaluated in this article, or claim that may be made by its manufacturer, is not guaranteed or endorsed by the publisher.

## Supplementary Material

Supplementary Figure 1 Expression of intrinsic cell death genes. Ethanol did not enhance levels of cell death associated genes caspase-3, caspase-8, and TRAIL were measured by RT-PCR.

Supplementary Table 1 GSEA analysis of inflammatory genes in males (EtOH v Control).

Supplementary Table 2 GSEA analysis of inflammatory genes in females (EtOH v Control).
