# Supplementary figures and images for: Chronic Ethanol Causes Persistent Increases in Alzheimer’s Tau Pathology in Female 3xTg-AD Mice: A Potential Role for Lysosomal Impairment
Source: Front Behav Neurosci. 2022 May 11;16:886634. doi: 10.3389/fnbeh.2022.886634 (PMC9131098; doi:10.3389/fnbeh.2022.886634)

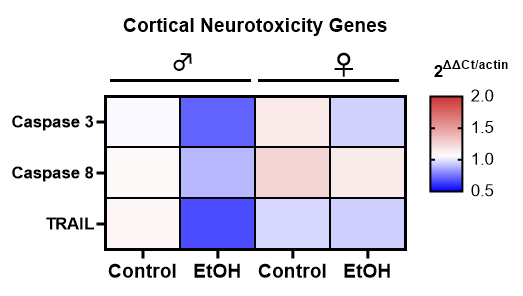

Supplement: Supplementary Figure 1 — Expression of intrinsic cell death genes. Ethanol did not enhance levels of cell death associated genes caspase-3, caspase-8, and TRAIL were measured by RT-PCR. [file Image_1.tif]
