# Supplementary material for: Chronic Ethanol Causes Persistent Increases in Alzheimer’s Tau Pathology in Female 3xTg-AD Mice: A Potential Role for Lysosomal Impairment
Source: Front Behav Neurosci. 2022 May 11;16:886634. doi: 10.3389/fnbeh.2022.886634 (PMC9131098; doi:10.3389/fnbeh.2022.886634)
Supplement: Supplementary Table 1 — GSEA analysis of inflammatory genes in males (EtOH v Control). [file Data_Sheet_3.DOCX]

**Supplemental Table 1: GSEA analysis of inflammatory genes in males (EtOH v Control)**

|  | **SYMBOL** | **RANK IN GENE LIST** | **RANK METRIC SCORE** | **RUNNING ENRICHMENT SCORE** | **CORE ENRICHMENT** |
| --- | --- | --- | --- | --- | --- |
| 1 | TLR7 | 0 | 0.473 | 0.1143 | Yes |
| 2 | TNF | 1 | 0.229 | 0.1695 | Yes |
| 3 | PPARG | 2 | 0.071 | 0.1866 | Yes |
| 4 | IFNA1 | 3 | 0.036 | 0.1952 | Yes |
| 5 | IFNB1 | 4 | 0.015 | 0.1988 | Yes |
| 6 | IFNG | 5 | -0.016 | 0.2026 | Yes |
| 7 | IL6 | 6 | -0.072 | 0.2199 | Yes |
| 8 | IL1B | 7 | -0.219 | 0.2728 | Yes |
| 9 | TGFB1 | 8 | -0.235 | 0.3295 | Yes |
| 10 | IRF3 | 9 | -0.337 | 0.411 | Yes |
| 11 | CD68 | 10 | -0.409 | 0.5097 | Yes |
| 12 | TLR4 | 11 | -0.877 | 0.7215 | Yes |
| 13 | B2M | 12 | -1.154 | 1 | Yes |

**Supplemental Table 2: GSEA analysis of inflammatory genes in females (EtOH v Control)**

|  | **SYMBOL** | **RANK IN GENE LIST** | **RANK METRIC SCORE** | **RUNNING ENRICHMENT SCORE** | **CORE ENRICHMENT** |
| --- | --- | --- | --- | --- | --- |
| 1 | IRF3 | 0 | 0.927 | 0.1646 | Yes |
| 2 | PPARG | 1 | 0.602 | 0.2713 | Yes |
| 3 | IFNB1 | 2 | 0.524 | 0.3642 | Yes |
| 4 | IL6 | 3 | 0.499 | 0.4528 | Yes |
| 5 | TLR7 | 4 | 0.494 | 0.5404 | Yes |
| 6 | IFNA1 | 5 | 0.411 | 0.6133 | Yes |
| 7 | IFNG | 6 | 0.402 | 0.6847 | Yes |
| 8 | IL1B | 7 | 0.398 | 0.7554 | Yes |
| 9 | TGFB1 | 8 | 0.335 | 0.8149 | Yes |
| 10 | TLR4 | 9 | 0.186 | 0.8478 | Yes |
| 11 | CD68 | 10 | 0.185 | 0.8806 | Yes |
| 12 | B2M | 11 | -0.147 | 0.9066 | Yes |
| 13 | TNF | 12 | -0.526 | 1 | Yes |
