# Supplementary material for: Chronic Ethanol Causes Persistent Increases in Alzheimer’s Tau Pathology in Female 3xTg-AD Mice: A Potential Role for Lysosomal Impairment
Source: Front Behav Neurosci. 2022 May 11;16:886634. doi: 10.3389/fnbeh.2022.886634 (PMC9131098; doi:10.3389/fnbeh.2022.886634)

Full Length Western Blots

Tucker et al 2022

Figure 3A: Total Tau in female cortex

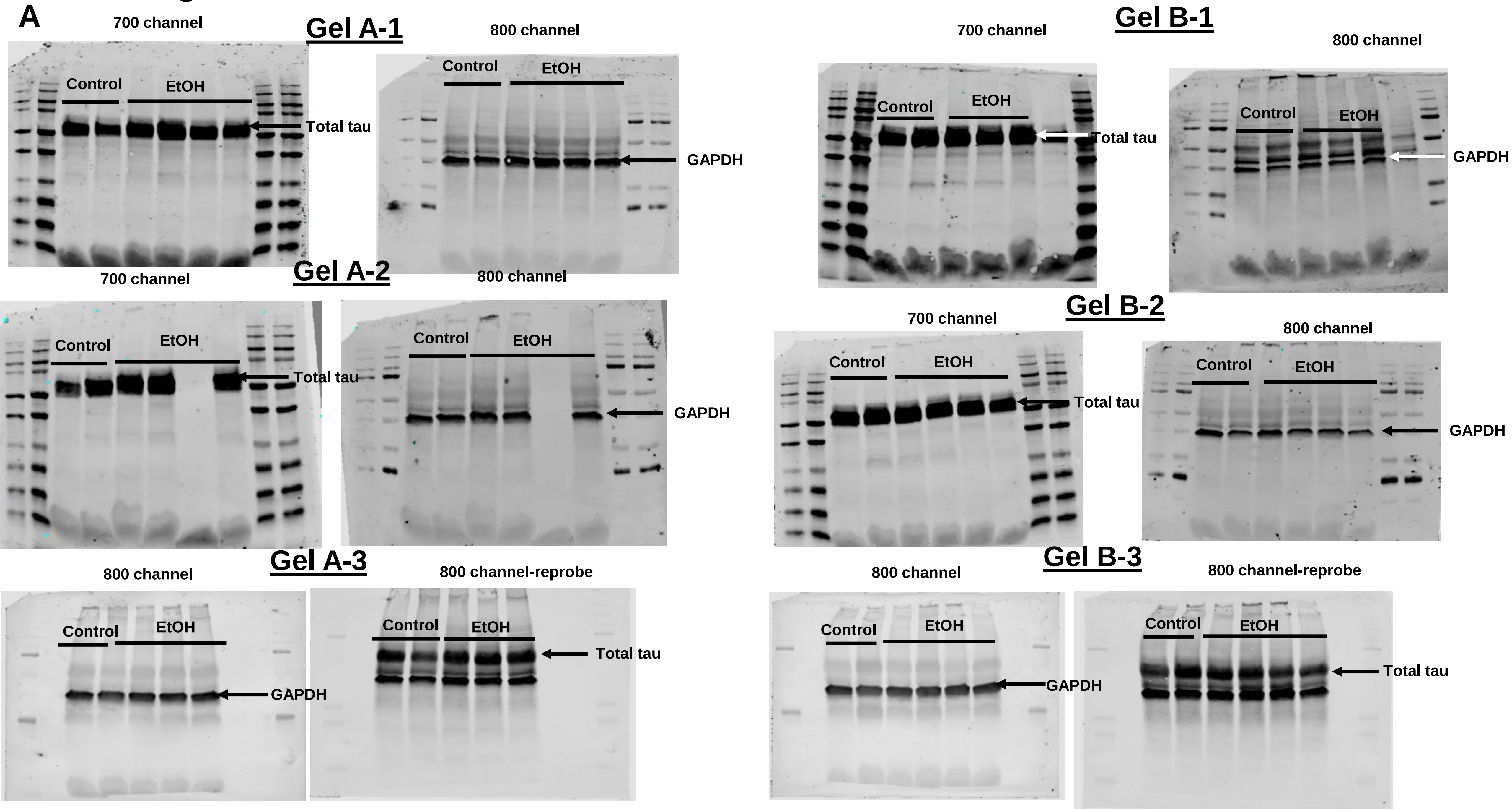

Figure 3B: p-tau214 in female cortex

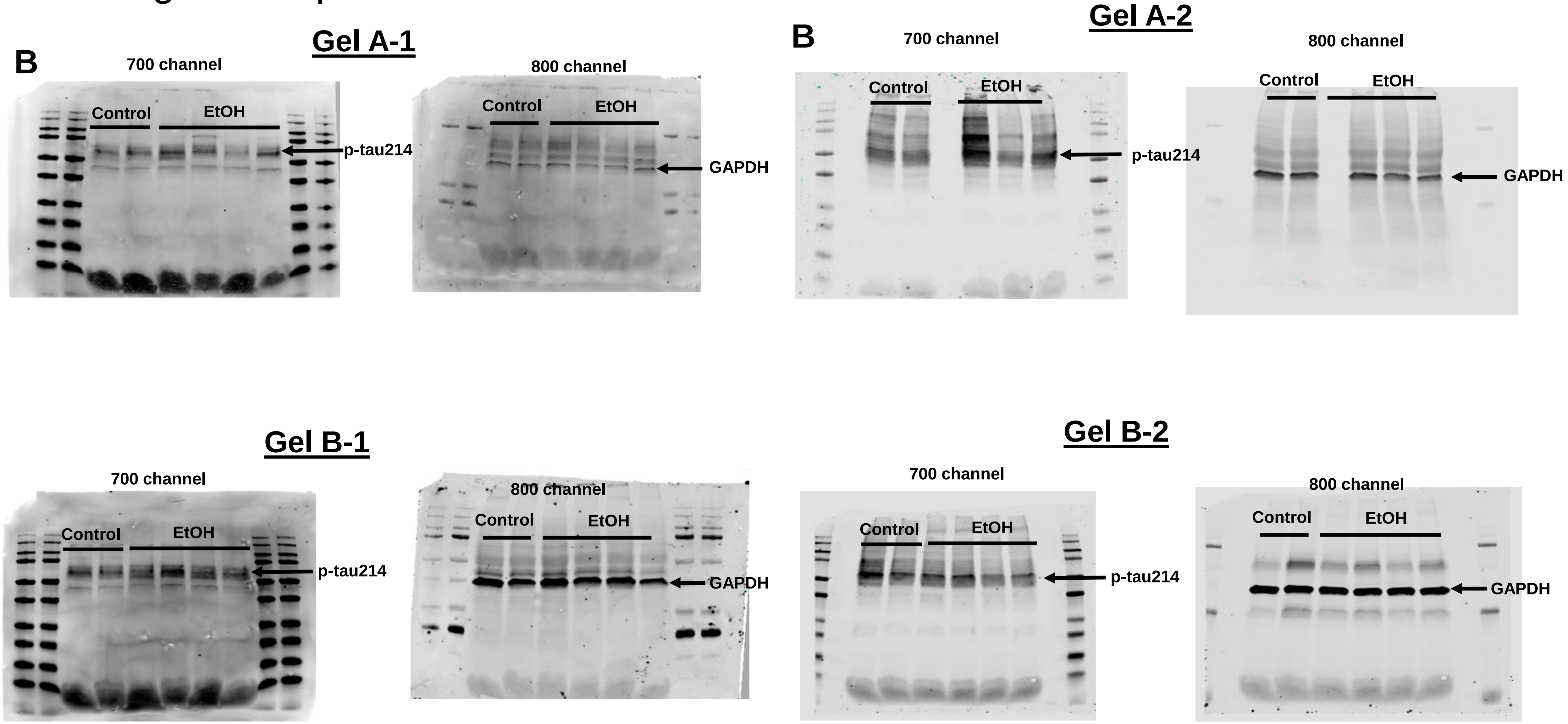

Figure 3C: Total Tau in female Hippocampus

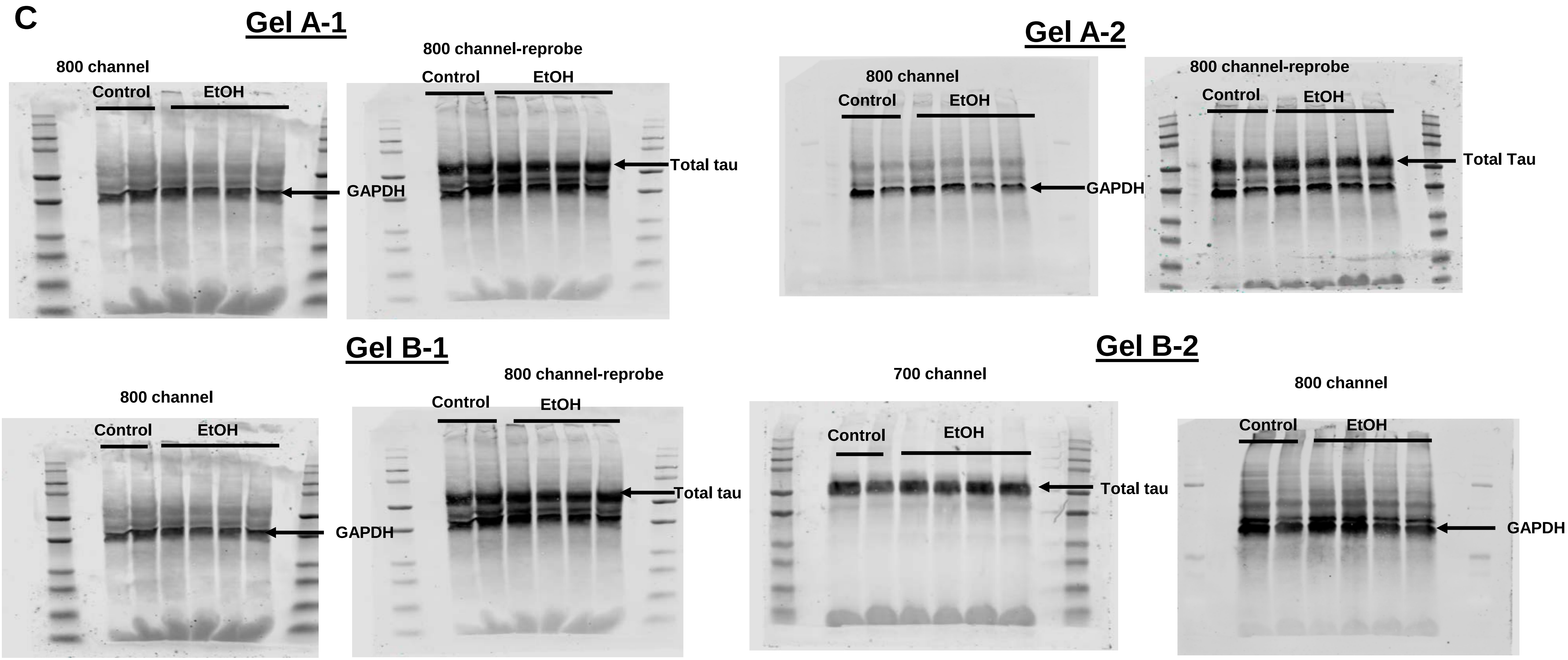

Figure 3D-E: pTau214 in female hippocampus and amyloid in female cortex

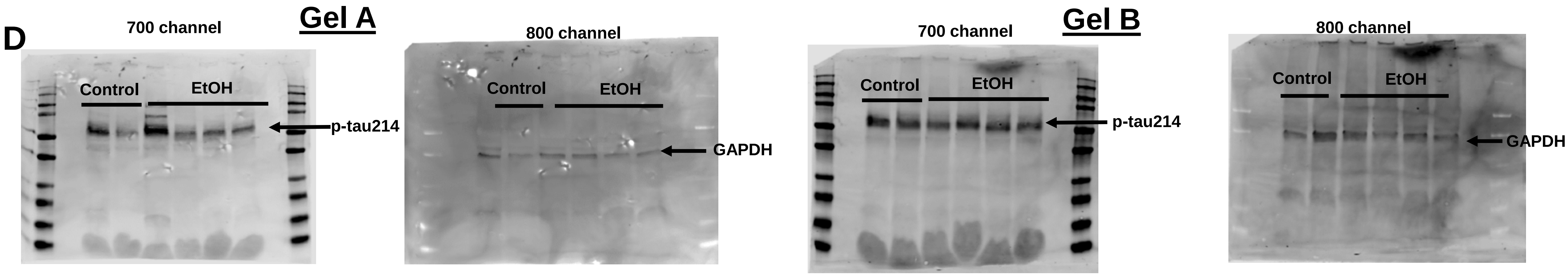

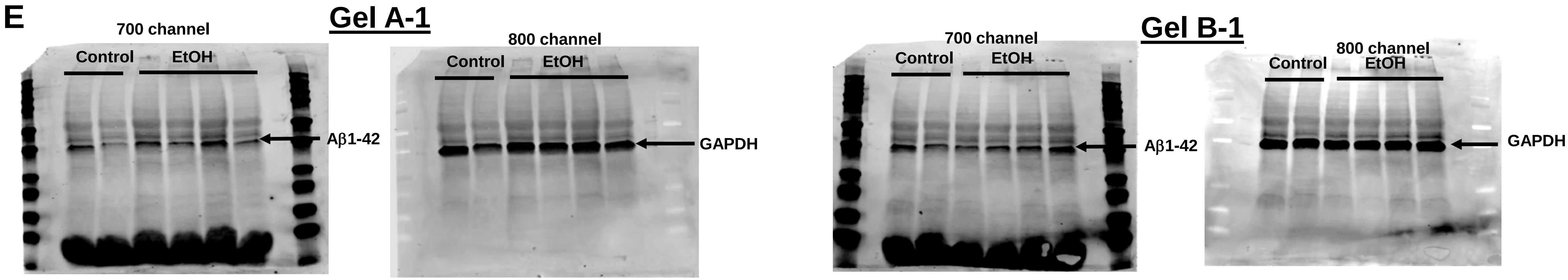

Figure 3F: Aβ1-42 in female Hippocampus

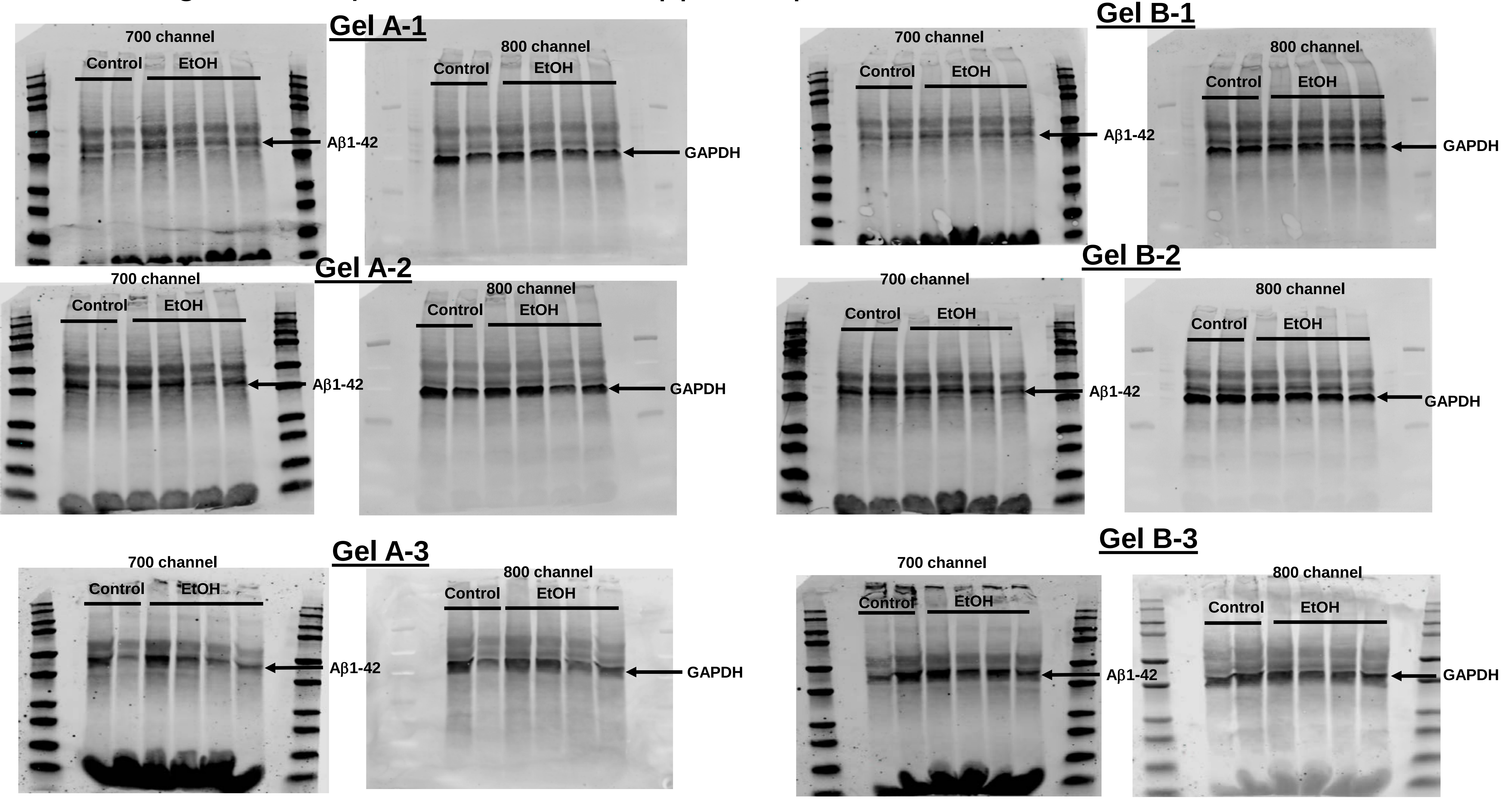

Figure 4A,B,E: Tau and amyloid in male cortex

A

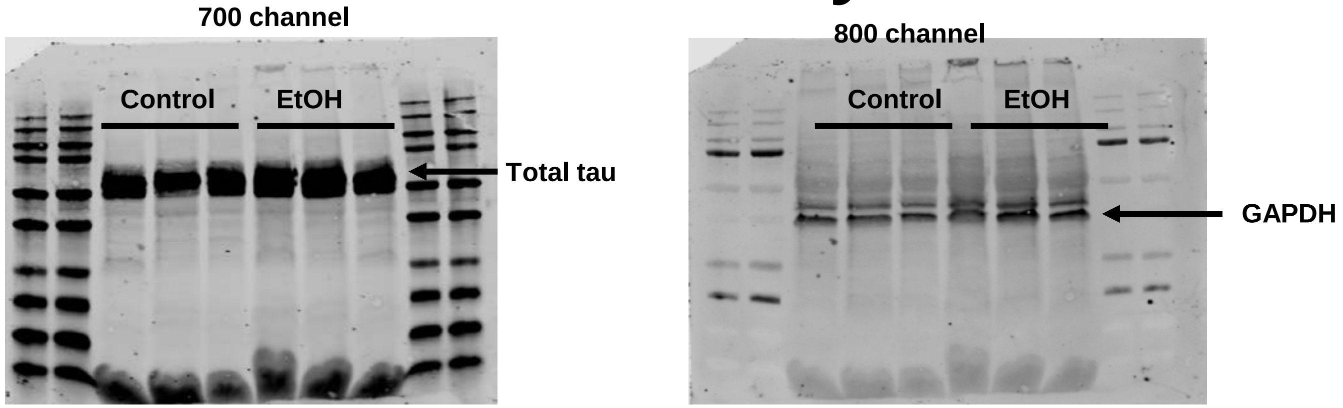

B

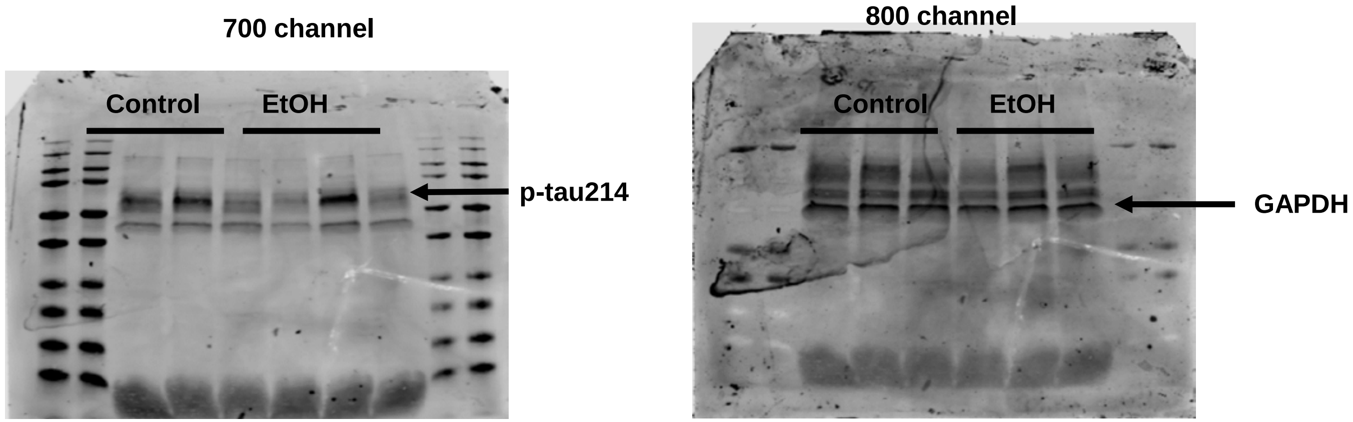

E

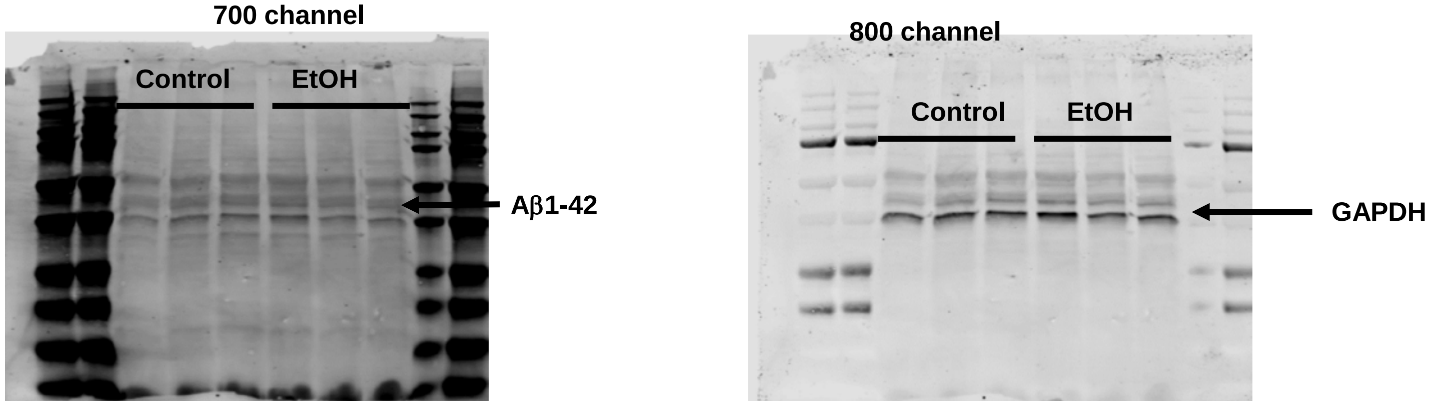

Figure 4C-D: Total Tau and pTau 214 in male hippocampus

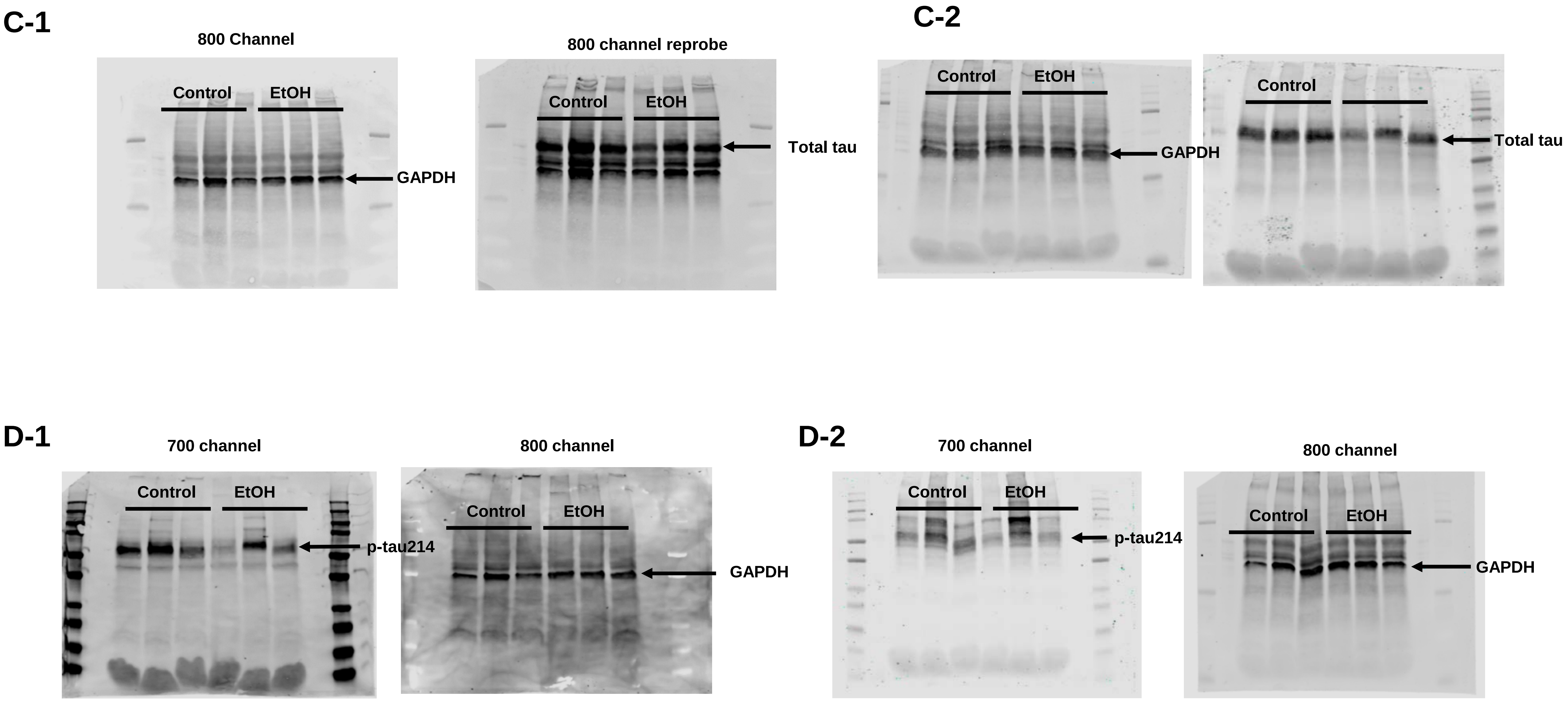

Figure 4F: Aβ1-42 in male hippocampus

F

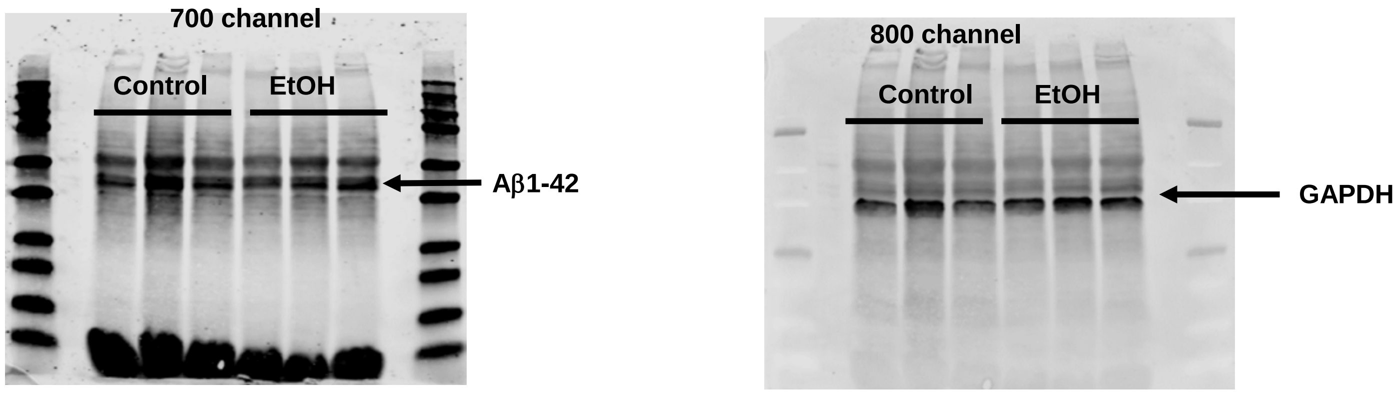

Figure 6A: Total GSK3β in female cortex

A

Gel A-cortex

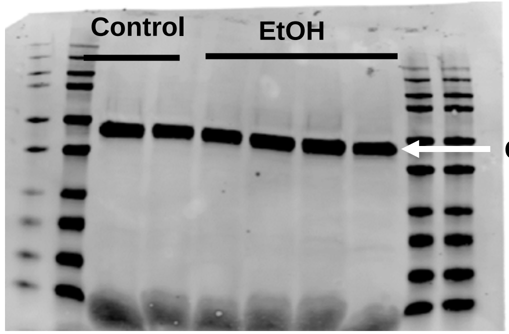

GSK3β

reprobe

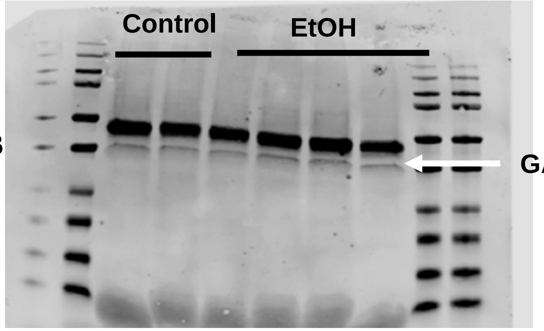

GAPDH

Gel B-cortex

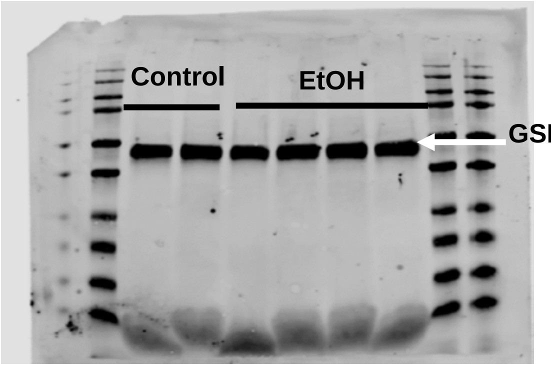

GSK3β

reprobe

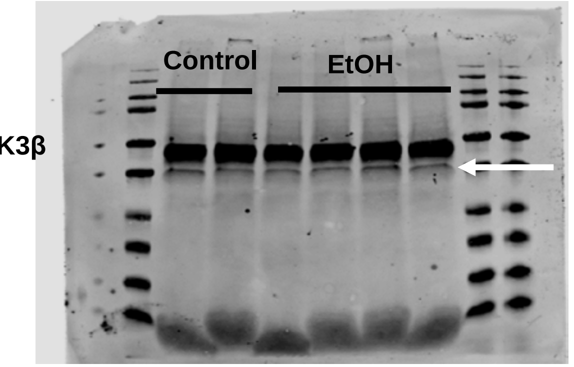

GAPDH

A

Gel A-hippocampus

700 channel

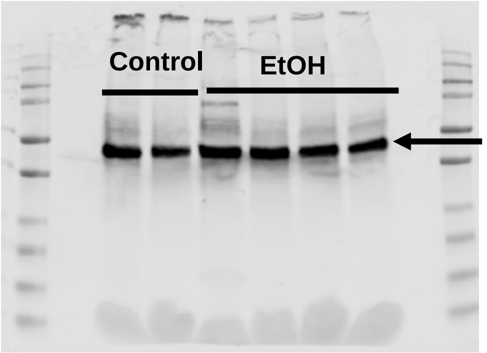

GSK3β

800 channel

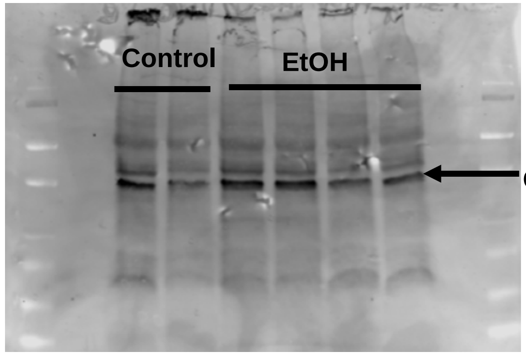

GAPDH

Gel B-hippocampus

700 channel

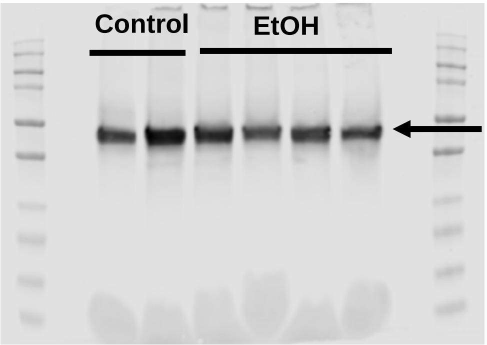

GSK3β

800 channel

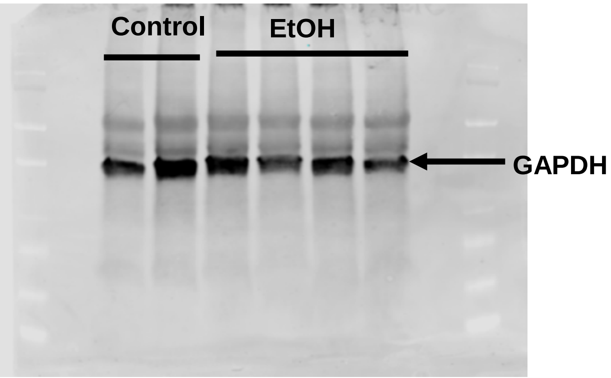

GAPDH

Figure 6B: GSK3β isoforms in female cortex

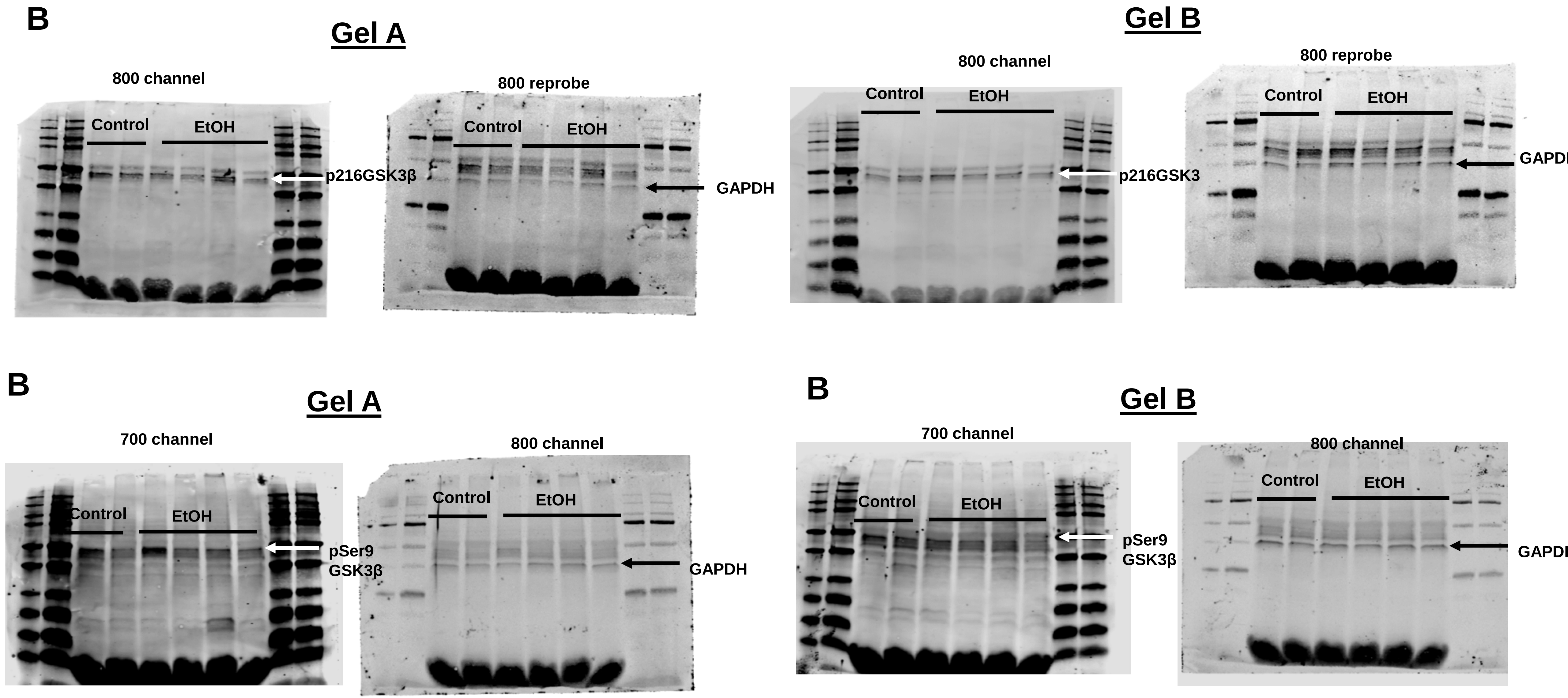

Figure 6C-D: PKA and PP2A in female cortex

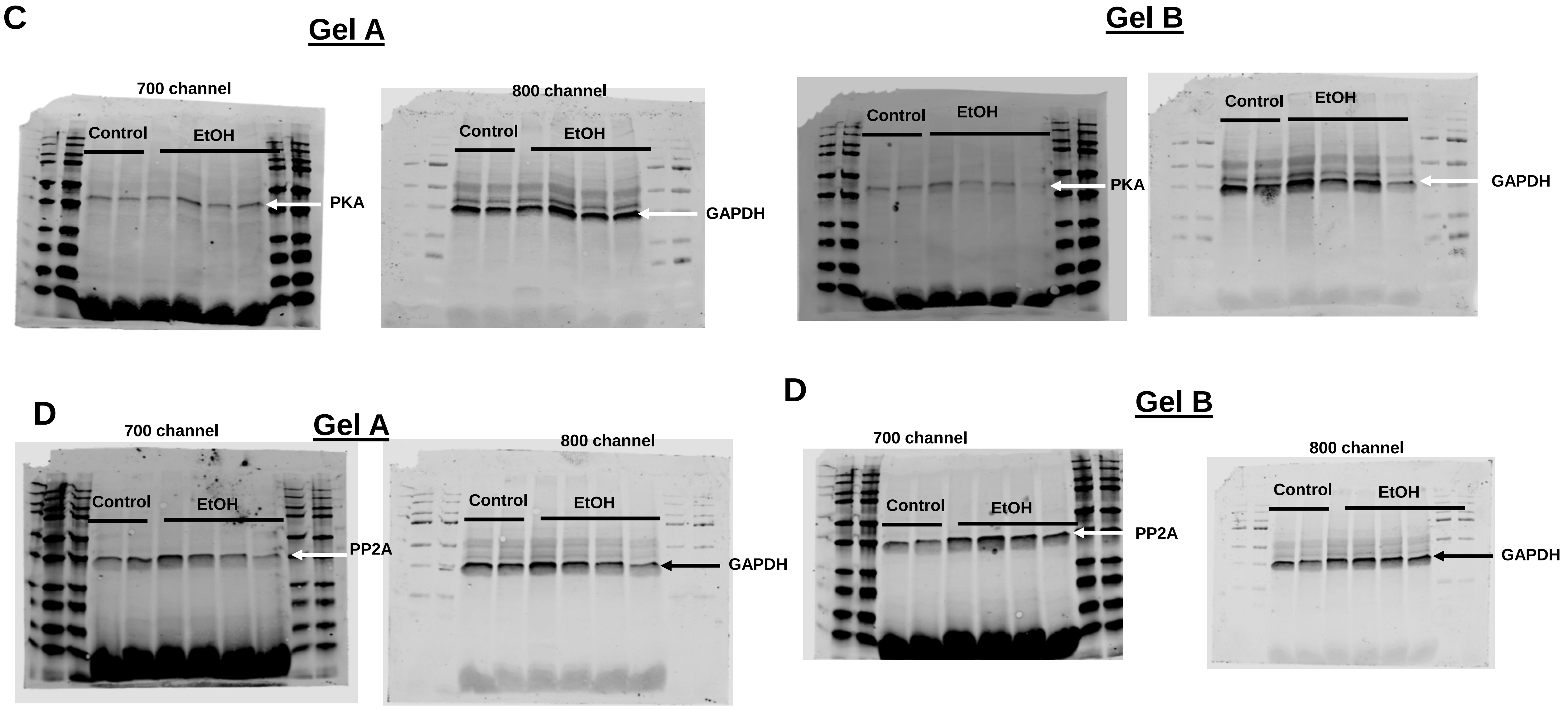

Supplement: Supplementary file 3 [file Data_Sheet_1.pdf]
